# Closing the evidence gap: why childhood cancer research must include fathers

**DOI:** 10.3389/phrs.2026.1610147

**Published:** 2026-08-12

**Authors:** Peter Francis Raguindin, André Oscar von Bueren, Gisela Michel

**Affiliations:** 1 Faculty of Health Sciences and Medicine, University of Lucerne, Lucerne, Switzerland; 2 CANSEARCH Research Platform for Pediatric Oncology and Hematology, Faculty of Medicine, Department of Pediatrics, Gynecology and Obstetrics, University of Geneva, Geneva, Switzerland; 3 Division of Pediatric Oncology and Hematology, Department of Women, Child and Adolescent, University Geneva Hospitals, Geneva, Switzerland

**Keywords:** childhood cancer, family support, father involvement, pediatric oncology, psychosocial care

## Introduction

A childhood cancer diagnosis affects the entire family. Each family member experiences the diagnosis differently, faces unique challenges, and develops their own ways of coping. While many studies have addressed parents (focusing on mothers) and siblings, fathers have been underrepresented in most research [1]. In many families, caregiving roles become divided after diagnosis. Mothers may take on more care tasks, while fathers may carry the financial responsibility [2]. At the same time, fathers may also try to maintain employment, while being present for medical decisions and the emotional needs of the ill child, other children, and their partner [2]. As a result, fathers may thus feel torn between multiple competing demands [2].

Beyond these practical demands, fathers also face substantial emotional challenges that remain underrecognized in the literature. Studies have shown that fathers suppress psychological distress because they believe they must be resilient for the child, partner, and family [3, 4]. While this emotional restraint may help fathers cope in the short term, it can also contribute to feelings of isolation and delayed emotional responses. However, empirical findings remain limited.

A systematic review by Olsavsky, et. al. highlighted the need for more studies on fathers [5]. For this commentary, we build on these recommendations and argue further that a) the limited inclusion of fathers in research is not merely an evidence gap. It also leads to (b) non-generalizable evidence on fathers, c) the development of support strategies not tailored or appealing to fathers, and d) implementation research that fails to adequately represent fathers’ experiences and perspectives.

## Evidence gap due to lack of studies on fathers

Studies of parents of children with chronic illness, including cancer, continue to enroll substantially fewer fathers than mothers [5, 6], which may be related to methodological and logistical barriers to their participation. Across literature, females are more likely to participate in surveys than males. Fathers often remain engaged in full-time employment [6] and may therefore be less available during routine daily care, being present only when important medical decisions are required. Even when present, fathers may prioritize spending their limited time with their child rather than participating in research activities [6]. Also, they may have fewer opportunities for direct contact with researchers or healthcare professionals who recruit participants. Some families may perceive that the mother’s response to a questionnaire already represents the family view [7]. Researchers may also unintentionally reinforce this pattern when study information is addressed broadly to “parents”, but communication and follow-up are conducted primarily through mothers as the main point of contact. As a result, fathers remain underrepresented in research.

## Underrepresentation leads to non-generalizable evidence

Because fathers are underrepresented in most surveys on families coping with childhood cancer, the psychosocial outcome of the childhood cancer experience remains largely unknown, or not generalizable for fathers. Their experience has been documented by some qualitative studies, yet very few quantitative studies or surveys have systematically targeted fathers as a study population. As a result, the existing findings, although scientifically valid, cannot be generalized to fathers. A review of psychosocial interventions for families of children with cancer found that fathers experience severe distress and would benefit from psychological interventions, yet remain largely underrepresented in studies on distress management [8].

## Support strategy for families may not appeal to fathers

Therefore, the support preferences of fathers have not been systematically assessed or integrated into the design of support interventions. A systematic review identified eleven psychosocial intervention programs for parents of children with cancer, built on cognitive-behavioral, systemic, and counselling models of change [9]. Some programs were developed for and tested exclusively with mothers of newly diagnosed childhood cancer [9]. In other words, “support for parents” has, in practice, often meant support designed around and validated with mothers. The scientific basis of these programs is well justified based on the literature. However, because fathers were not part of the development or testing of these programs, effectiveness of such interventions may not be readily transferable to fathers.

## Trials and implementation research does not represent fathers

Another systematic review in 2025 [10] pooled trials of psychological interventions delivered to family members of pediatric cancer patients and found that these interventions improved resilience, with benefits sustained into the short to medium term. Although this is an encouraging result, the analysis reported outcomes for “family members” as a single pooled category rather than separately for fathers and mothers, so it cannot tell us whether the beneficial effect would be similar for fathers, who typically make up a small minority of trial samples [10]. Newer randomized trials of technology-based psychological support for parents of children with cancer follow the same pattern, reporting a single combined parent outcome rather than breaking results out by father or mother status [11]. Until fathers are adequately represented in clinical trials and their outcomes are analyzed and reported as a distinct group, evidence on the effectiveness of interventions specifically for fathers will remain limited.

## Towards a more complete picture of family adjustment and family support

Overall, these limitations describe a major underlying problem. Research on families during childhood illness, particularly in cancer, has developed largely without systematic inclusion of fathers [5]. A more accurate model of family adjustment to childhood cancer requires data from both parents, collected and analyzed in a manner that does not treat one parent’s report (that is the mother) as a proxy for the family unit.

Future studies should be designed to include fathers from the beginning [6]. Recruitment should identify fathers directly, not only through mothers [12]. Study invitations should name fathers explicitly and explain why their perspective is needed, even when the mother has already participated [7, 12]. Researchers should offer flexible participation options, shorter online surveys, telephone interviews, and short modular questionnaires, all have been suggested to increase participation [12]. Recruitment should also occur through multiple points of contact, including clinics, registries, psychosocial teams, and parent organizations, to provide more opportunities for fathers to participate [12].

Current support measures also need to reflect fathers’ experiences more accurately. Studies should assess depression, anxiety, distress, fear of recurrence, work strain, couple relationship quality, social support, caregiving roles, and unmet needs. They should also capture forms of coping and distress that may be less visible, such as emotional suppression, work-based coping, difficulty asking for help and feelings of inadequacy. Whenever possible, studies should include both parents and examine similarities and differences within families and between genders. This would clarify how fathers’ and mothers’ distress interact, how roles are negotiated, and how support can be offered to fit individual preferences.

The next step is not to replace the focus on mothers, but to complete the family picture by strengthening the evidence on fathers. Childhood cancer care is strongest when it understands how each family member is affected and how family roles shape adjustment. Fathers should be included in research designs, measured with appropriate tools, and addressed in clinical support pathways. Future work should generate estimates of the prevalence of fathers’ psychological distress, identify fathers at higher risk, and evaluate whether targeted or family-based interventions improve outcomes. Without these steps, fathers will remain visible in everyday family life but continue to be overlooked in both research evidence and clinical practice.
